# Immunometabolic Modulatory Role of Naltrexone in BV-2 Microglia Cells

**DOI:** 10.3390/ijms22168429

**Published:** 2021-08-05

**Authors:** Natalia Kučić, Valentino Rački, Roberta Šverko, Toni Vidović, Irena Grahovac, Jasenka Mršić-Pelčić

**Affiliations:** 1 Department of Physiology and Immunology, Faculty of Medicine, University of Rijeka, Braće Branchetta 20, 51000 Rijeka, Croatia; 2 Department of Neurology, Clinical Hospital Center Rijeka, University of Rijeka, Krešimirova 42, 51000 Rijeka, Croatia; valentino.racki@uniri.hr; 3 Emergency Department, Clinical Hospital Center Rijeka, University of Rijeka, Krešimirova 42, 51000 Rijeka, Croatia; roberta.sverko@gmail.com (R.Š.); vidovic.toni@yahoo.com (T.V.); 4 Pharmacy Irena Grahovac, Trg I. Istarske brigade 5, 52100 Pula, Croatia; irena.grahovac@pu.t-com.hr; 5 Department of Pharmacology, Faculty of Medicine, University of Rijeka, Braće Branchetta 20, 51000 Rijeka, Croatia; jasenka.mrsic.pelcic@medri.uniri.hr

**Keywords:** naltrexone, BV-2 microglia, immunometabolism, drug repurposing

## Abstract

**Background**: Naltrexone is an opioid receptor antagonist commonly used to treat opioid and alcohol dependence. The use of low dose naltrexone (LDN) was found to have anti-inflammatory properties for treatment of diseases such as fibromyalgia, Crohn’s disease, multiple sclerosis and regional pain syndromes. Related to its anti-neuroinflammatory properties, the mechanism of action is possibly mediated via Toll-like receptor 4 antagonism, which is widely expressed on microglial cells. The aim of the present study was to assess the immunometabolic effects of naltrexone on microglia cells in in vitro conditions. Methods: All experiments were performed in the BV-2 microglial cell line. The cells were treated with naltrexone at 100 μM concentrations corresponding to low dose for 24 h. Cell viability was assessed for every drug dose. To induce additional activation, the cells were pretreated with LPS and IFN-γ. Immunofluorescence was used to analyse the classical microglial activation markers iNOS and CD206, while Seahorse was used for real-time cellular metabolic assessments. mTOR activity measured over the expression of a major direct downstream target S6K was assessed using western blot. Results: LDN induced a shift from highly activated pro-inflammatory phenotype (iNOS^high^CD206^low^) to quiescent anti-inflammatory M2 phenotype (iNOS^low^CD206^high^) in BV-2 microglia cells. Changes in the inflammatory profile were accompanied by cellular metabolic switching based on the transition from high glycolysis to mitochondrial oxidative phosphorylation (OXPHOS). LDN-treated cells were able to maintain a metabolically suppressive phenotype by supporting OXPHOS with high oxygen consumption, and also maintain a lower energetic state due to lower lactate production. The metabolic shift induced by transition from glycolysis to mitochondrial oxidative metabolism was more prominent in cells pretreated with immunometabolic modulators such as LPS and IFN-γ. In a dose-dependent manner, naltrexone also modulated mTOR/S6K expression, which underlies the cell metabolic phenotype regulating microglia immune properties and adaptation. Conclusion: By modulating the phenotypic features by metabolic switching of activated microglia, naltrexone was found to be an effective and powerful tool for immunometabolic reprogramming and could be a promising novel treatment for various neuroinflammatory conditions.

## 1. Introduction

Naltrexone is a clinically approved drug and the purpose of the present study was to investigate the utility of this drug under the drug repurposing concept ‘an old molecule for new therapeutic approach’. As a semi-synthetic opioid with competitive antagonist activity at mu opioid receptors, naltrexone is traditionally used in clinical practice for the treatment of alcohol and opioid dependence [1]. More recently, its indications have been expanded for use in various inflammatory conditions for which the drug is prescribed at lower doses such as fibromyalgia, Crohn’s disease, multiple sclerosis and regional pain syndromes [2,3,4]. Low-dose naltrexone (LDN) has a very diverse range of effects on cells by differently acting on gene expression at standard doses [5]. This diversity and dose-dependent impact of naltrexone has created difficulties in establishing a unique mechanism of action and optimal therapeutic approach. Thus, following initial medical use, the list of potential characteristics of LDN in biomedicine is growing, leading to its use as an immune modulator [6] with anti-inflammatory properties [2,7]. Moreover, it is also considered as a ‘glial attenuator’, being able to minimize the pro-inflammatory response of activated glia cells [8] by modulating Toll-like receptor 4 signaling [9,10,11].

Microglia are immune cells of the brain that act as surveying sensors with self-adaptive effector properties in response to brain pathophysiology [12,13,14], and as such, they play a key role in neuroinflammatory diseases. Likewise, considering them as the resident macrophages of the brain, microglia are capable of being transformed from the resting to reactive macrophage-like state and thereby share an inflammatory and metabolic signature of activated macrophages [15,16]. Depending on the environmental conditions, microglia/macrophages undergo specific changes through which they shift from M1, the classically activated pro-inflammatory phenotype, to M2, the alternatively activated anti-inflammatory phenotype [17,18,19]. Recent research has shown that microglial function in in vivo conditions is more complex than the arbitrary M1/M2 classification and is more dependent on their location and the environment [20,21]. During microglia/macrophage activation, cellular metabolic reprogramming occurs, playing a key role in phenotype switching [22]. This process helps the cell appropriately respond to new requirements. Such acquired immunometabolic phenotype(s) are more complex than the M1/M2 model [23,24]. Therefore, additional understanding of how metabolic disorders and modulation of microglia are related to their immune functionality is required, which gives a new therapeutic approach in treatment of neurological diseases by interfering with the immunometabolic cell profile [25]. However, it is important to point out that the M1/M2 dichotomy can be observed in controlled in vitro conditions, and BV-2 microglia cells respond to stimulation by LPS and IFN-γ, which is similar to primary microglia [24]. The use of BV-2 cells presents a limitation that is present in many immortalized cell lines [26]. Furthermore, we use the metabolic status to assess microglial function and changes in pro- or anti- inflammatory profiles, as it can be complementary to markers to indicate their overall functional status [24].

Upon stimulation, activated immune cells undergo a metabolic switch to meet the demands of cells by regulating the balance between inflammatory and regulatory immune phenotypes [27]. A metabolic switch implies cell adaptation through ‘metabolic checkpoints’, which sense metabolic status and identify availability of specific metabolites used for immune cell signaling responses [28]. Additionally, metabolism contributes to development of the immune profile by directly impacting its bioenergetic configuration in order to balance its activated properties [27]. In this regard, mitochondria are critical links between metabolic features and immune specificities underlying cell phenotype and function [29]. However, mitochondria are not just energy production sites, they are also dynamic modulators of both cell physiology and immunity [30] and, as such, studying mitochondrial physiology is a great challenge in the field of immunometabolism.

Mammalian target of rapamycin (mTOR) is a central metabolic sensor and metabolic integrator that mediates cell signaling events [31]. The mTOR-mediated translational program in configuring metabolism is accompanied by substantial changes that support anabolic pathways to adopt metabolic plasticity underlying immune cell activation, and thereby, shaping the immune response [32,33]. Given its roles as an energy sensor and a metabolic hub, mTOR has been implicated in regulating responses related to metabolic status. Furthermore, as a master regulator of energy homeostasis, its fundamental role is in integrating different metabolic pathways, including glycolysis and oxidative phosphorylation (OXPHOS), by positively modulating mitochondrial biogenesis and energetical activity [27,28,32,33,34].

In this study, we investigated drug repositioning for naltrexone through its modulatory effect on microglial activation using BV-2 cells. We hypothesized that the naltrexone-driven microglia metabolic program influences immune phenotypic polarization, and could be considered as a novel approach in treatment of neuroinflammatory diseases. We introduced naltrexone as a major player of microglia deactivation leading to a beneficial anti-inflammatory phenotype based on a metabolic shift by the cell proteomic balance in maintaining mTOR activity. Hence, the molecular metabolomics’ targets as modifiers of cellular energy homeostasis gain a promising therapeutic significance in terms of guidelines for improving potential therapeutic molecular targets by which naltrexone modifies metabolic mechanism(s) in neuroinflammatory conditions.

## 2. Results

### 2.1. Naltrexone Induces an Anti-Inflammatory M2 Polarization State in BV-2 Cells

**Figure 1:**
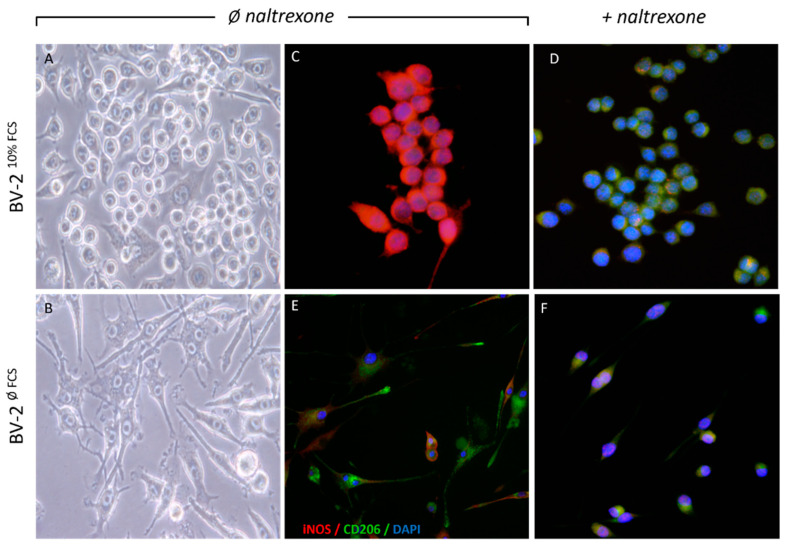
A distinct cell morphology and M1/M2 activated state of BV-2 microglia cells under the different culture conditions and the effect of naltrexone in the both cell phenotypes. The cells were cultivated long term in two different conditions: in the DMEM medium supplemented with 10% FCS (BV-2^10%FCS^) (upper panel) or without FCS (BV-2^øFCS^) (lower panel) for 96 h prior to experiments. Different cell morphology phenotypes (amoeboid, (**A**) and branched, (**B**)) are shown and clearly visible with the bright field /phase-contrast (left panel) and immunofluorescence (IF) (right panel) images. BV-2^10%FCS^ cells not treated with naltrexone (**C**) exhibit increased iNOS expression, without visible CD206 expression, while the opposite is seen in cells treated with naltrexone (**D**). There are no significant changes in marker expression seen in BV-2^øFCS^ with or without naltrexone stimulation (**E**,**F**). The IF co-localization images (**C**–**F**) are merged using the M1 cytoplasmic marker iNOS (red fluorescence, labelled with anti-rabbit Ab conjugated with Alexa 555), the M2 cytoplasmic marker CD206 (green fluorescence, labelled with anti-rat Ab conjugated with Alexa 488) and the nucleic stain with DAPI (blue fluorescence). Image magnification was 40–60×.

For the purpose of testing naltrexone as an anti-inflammatory drug, we used a model of BV-2 cells that exhibits morphological and functional features of primary microglia and comparable reactive microglia. Namely, these cells spontaneously show a dual phenotype when cultivated in the standard conditions of DMEM supplemented with up to 10% *v*/*v* serum (10% fetal calf serum, FCS). First, we compared BV-2 cells in growing conditions with or without serum. After growing in 10% DMEM for 96 h (BV-2^10%FCS^), they reside in the culture in dominantly ameboid form. Differences in cell shape are clearly visible as BV-2^10%FCS^ cells retain an ameboid round blob/macrophage-like morphology, while BV-2^øFCS^ shift to ramified/branched/spider-like morphology (Figure 1A,B). We found that these cell cultures always contain all morphologies but change predominantly towards a certain type depending on stimulation. Herein the minor parts of the cells in the transitional phase of the cultivation period remain in the bipolar/“rod-like” form, but they are nevertheless mostly predominantly branched/“spider-like”. Other research has shown that these bipolar cells more closely resemble an anti-inflammatory phenotype or a reconstitutional phenotype in traumatic brain injury or penumbra in ischemic stroke [35]. Next, we determined the level of M1/M2 cytoplasmic marker proteins’ expression. The results of the immunofluorescence point to a constitutively high expression of iNOS, a distinct activation marker in BV-2^10%FCS^ (Figure 1C), indicating increased NO production and associated M1-like cell activation properties. We characterized this activated cellular phenotype as iNOS high positive (iNOS^high^). The BV-2^10%FCS^ phenotype constitutively exhibits low expression of the M2 anti-inflammatory marker protein CD206 (Figure 1C). Inclusively, the BV-2^10%FCS^ phenotype exhibits high expression of iNOS with low expression of CD206, and this expression pattern was specified as an activated cell profile (iNOS^high^CD206^low^).

After establishing growth conditions supportive for BV-2 activation, we treated BV-2^10%FCS^ cells with naltrexone at concentrations of 10, 50, 100, 250, 500, and 1000 μM to determine the drug effect on cell viability. An assessment of drug efficacy was performed based on preserved cell viability (>96%) and was optimal at doses up to 250 μM and greatly reduced at a dose of 500 μM and above. Thus, the most effective drug dose in terms of anti-inflammatory properties was 100 μM, corresponding to low dose naltrexone (LDN) [2]. In our cell model, negative to low iNOS and highly expressed CD206 as a proteomic pattern occurred in LDN-treated cells after 24 h upon drug administration, associated with a quiescent cell phenotype (iNOS^low^CD206^high^) (Figure 1D). Accordingly, a distinctive feature of the quiescent cell profile (iNOS^low^CD206^high^) was previously achieved by removing the FCS from the cultivation media through the time course of 96 h (BV-2^øFCS^). The resting states most likely arose from suppressing their constitutive activation in serum free-media and inducing a shift in cell morphology to a more ramified state (Figure 1 lower panel). Naltrexone had no major effect on constitutively expressed markers in quiescent cells (Figure 1F) compared to control (Figure 1E). At this stage, the phenotypic polarization state of BV-2 microglia cells under the influence of naltrexone was closely defined based on their activation state.

### 2.2. Naltrexone Induces an Immunometabolically-Suppressive Phenotype in BV-2 Microglia Cells

**Figure 2:**
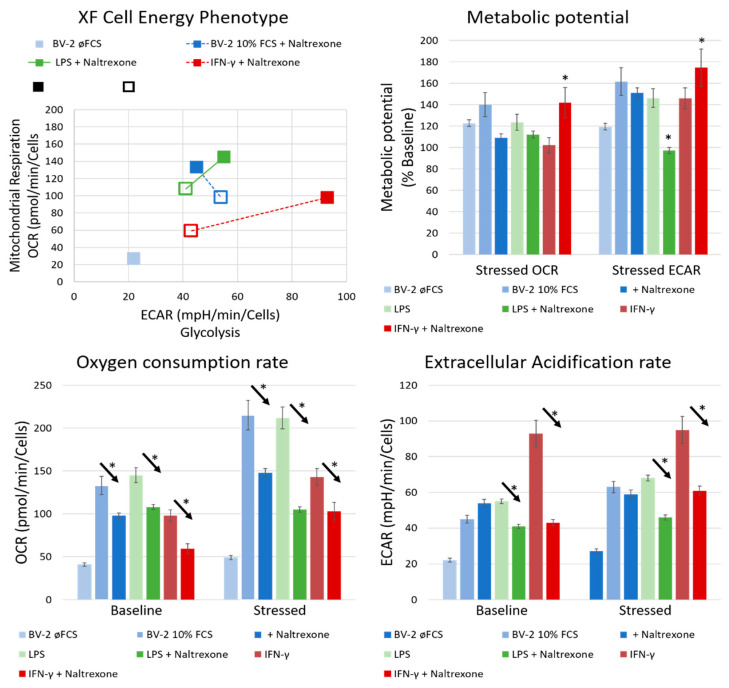
Naltrexone suppresses the highly metabolically activated BV-2 microglia cells. Cell energy phenotype based on real-time metabolism assay of BV-2 microglial cells with the baseline (empty square) and stressed metabolic potential after stressing the cells with FCCP and oligomycin (filled square). Cells were cultivated for 96 h in distinct cultivated conditions and then pretreated with 10 nM LPS and 100 UI/mL of IFN-γ for 24 h prior to a 100 μM concentration of naltrexone administration. After 24 h, cell samples were analysed with XF Cell Energy Phenotype kit by Seahorse. Corresponding symbols are represented in colour. Statistical significance (*) was set at *p* ≤ 0.05. Arrows with the * symbol denote the statistically significant difference between two paired samples.

To further correlate the M1/M2 cell immune profile with their metabolic signature, we used a real-time metabolic assay to assess the energetic status of the cell (Figure 2).

The baseline metabolic potential of BV-2^øFCS^ cells was mostly quiescent with minimal metabolic activity (low OCR and ECAR), while the BV-2^10%FCS^ cells were glycolytically active (low OCR and high ECAR). Both cell phenotypes had an equal metabolic potential to respond to stimuli when exposed to stressors. Therefore, after stressing the cells with FCCP and oligomycin, there was a shift towards a more energetic state in both cell cultures, with a similar increase of metabolic potential. On the other hand, BV-2^10%FCS^ exhibited greater aerobic metabolic reserve.

In addition, stimulation with pro-inflammatory stimuli like microbial compounds and interferons contributes to macrophage/microglia polarization associated with an inherent specific proteomic [36] and metabolic profile [22], leading to distinct immune responses [16]. Therefore, we chose to examine BV-2 microglial cells’ responses to pro-inflammatory stimuli like lipopolysaccharide (LPS) and interferon-gamma (IFN-γ).

Particularly high metabolic activity was measured in BV-2^10%FCS^ cells additionally stimulated with LPS and IFN-γ (Figure 2), which modulated energy metabolism leading to a metabolic switch from baseline to glycolysis, promoting a glycolytic phenotype in classically M1-activated cells. Thereby, LPS+IFN-γ enhanced glycolytic flux and cells became highly glycolytic. The shift to glycolysis was much higher in IFN-γ- than in LPS-treated cells. Furthermore, IFN-γ induced an increase in glycolysis to a greater extent when compared to LPS, thus promoting aerobic metabolism accompanied by a decreased oxidative consumption rate. This metabolic shift was followed by a reduction in oxidative phosphorylation. In contrast, BV-2^øFCS^ cells were metabolically and energetically inactive.

LDN proved effective in reducing aerobic metabolism in FCS-/LPS-/IFN-γ-induced energetically activated cells with certain comparable differences in the ECAR response in our settings. Naltrexone abolished the glycolytic metabolic reprogramming of the cells activated by highly potent immunometabolic modulators such as LPS and IFN-γ. Naltrexone thus possesses metabolic balancing properties, reversing a hyperactive cellular state to a normal or even hypo-energetic one.

The immunometabolically suppressive phenotype of BV-2 microglia cells was based on the shift from the highly activated phenotype (iNOS^high^CD206^low^) accompanied by a metabolically glycolytic state (low OCR and high ECAR) to the quiescent anti-inflammatory M2 phenotype (iNOS^low^CD206^high^), maintaining a lower energetic state with low lactate production and high oxygen consumption (high OCR and low ECAR), supporting oxidative phosphorylation (OXPHOS).

### 2.3. Naltrexone Calibrates a Metabolic State in BV-2 Cells by Modulating the mTOR/S6K Pathway

The phenotypic characteristics of BV-2 cells in this model have so far been related to the M1/M2 polarization state based on their metabolic status.

**Figure 3:**
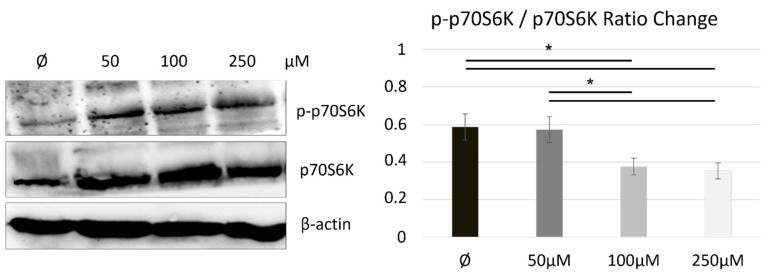
Immunoblot of p70S6K/p-p70S6K expression indicating a naltrexone/mTOR-dependent interrelation mediated by p70S6K expression and phosphorylation. Protein expression of the total p70 of the S6 kinase (p70S6K) subunit and its phosphorylation fraction p-p70S6K in untreated or naltrexone-treated BV-2^10%FCS^ cells is shown by Western blot as representative of three individual experiments (*n* = 3). Quantification analysis indicated statistical significance in the p70S6K expression and phosphorylation ratio change. (*) was set at *p* ≤ 0.05.

Cellular energy homeostasis is regulated by an energy-responsive molecule target of rapamycin (mTOR), which coordinates energy consumption and mitochondrial energy production by stimulating synthesis of mitochondria-related ribosomal proteins and metabolic enzymes [33], which contributes to modulation of the proteomic phosphorylation in the cell. To explore how mTOR, as an energy-responsive molecule in cell metabolic homeostasis, is modulated by naltrexone, we detected the expression of a major direct downstream target of the mTOR/S6 kinase (S6K) signaling pathway, ribosomal p70 S6 kinase (p70S6K), by using western blot. Figure 3 shows that administration of LDN increases total protein expression of p70S6K in a dose dependent manner. Naltrexone treatment led to an increase in the total cytoplasmic protein fraction of p70S6K and to a lesser extent to an increase in phosphorylation component p-p70S6K (Figure 3), suggesting co-nuclear transcription and protein synthesis on ribosomes for metabolic adaptation by maintaining biosynthesis in balance.

Although there was a trend towards increased mTOR p70S6K subunit expression in naltrexone-treated cells, the proportion of phosphorylated p-p70S6K protein fraction was only slightly altered. This gives us possible insight into the mechanism by which naltrexone modulates mTOR activation, which does not involve protein synthesis, at least not in its short-term low-dose application.

## 3. Discussion

In this study, we have tried to reveal new properties of an ‘old’ drug for its additional use, which is becoming an increasingly important approach in pharmacological research.

### 3.1. What Is the Importance of Studying Naltrexone as an Anti-Inflammatory and Immunomodulatory Drug?

Naltrexone, an opioid receptor antagonist, is classically used for medication-assisted treatment of alcoholism or opioid-use disorders, standardly prescribed in daily oral doses of at least 50 mg, depending on the addiction [37]. Although naltrexone has been shown to be effective in the treatment of these conditions, adherence to daily dosing and its severe side effects such as anxiety, depression, sleep disturbance, and irritability precluded its use long-term [3,37]. Recently, there have been several studies demonstrating beneficial effects of low-dose naltrexone as a potential treatment of various inflammatory conditions [3].

Low-dose naltrexone (LDN) was first introduced to the public in 1985 by B. Bihari [6], who observed a reduction in the level of multiple pro-inflammatory cytokines after the administration of the drug. In addition, LDN administration exerts qualitatively different pharmacodynamical effects in relation to the applied quantity [7].

Other studies have discovered similar findings. In a pilot study by Parkitny et al., 4.5 mg of naltrexone was administered each night to patients suffering from fibromyalgia. The treatment resulted in significant reduction in serum levels of proinflammatory cytokines, including interleukin (IL)-1, IL-2, IL-12, IL-18, interferon gamma (IFN-γ), granulocyte-macrophage colony-stimulating factor (GM-CSF), and TNF-α [38]. Due to its immunomodulatory effects, LDN has been already studied as a potential treatment for multiple sclerosis, Crohn’s disease, cancer, Hailey-Hailey disease, complex-regional pain syndrome and others [5,11,39].

Since the balance between protective and harmful effects of the immune system is based on the cytokines’ profile, which promotes or reduces inflammation, drugs with the properties of reducing the pro-inflammatory and promoting the anti-inflammatory response [10] could be effectively used for the treatment of neuroinflammatory diseases [37]. Thus, other than just reducing the pro-inflammatory cytokines, naltrexone in low doses ranging from 1 to 5 mg, acts as a glial modulator [7] by specifically binding to Toll-like receptor 4 (TLR-4) as an antagonist. Since TLR-4 is usually expressed on activated microglial cells, by binding to TLR-4, LDN could reduce neuroinflammation, and hence is referred to as a “glial attenuator” [2,7]. Microglial attenuation via LDN administration was confirmed in the present study as well. Considering these observations, LDN could potentially have a role in the treatment of various neuroinflammatory diseases.

Current research on the effects of naltrexone on microglial cells shows that a wide concentration range can be used to exert a beneficial anti-inflammatory effect in in vitro conditions [11]. Furthermore, both the (+)-naltrexone and (−)-naltrexone isomers affected the inhibition of LPS-induced NO production in BV-2 cells in a dose range from 0.1 μM to 1000 μM, with an IC50 of approximately 100 μM for both isomers [11]. We have used the (−)-naltrexone stereoisomer and shown that this isomer also has immunomodulatory properties, as was previously shown by Wang et al. [11].

Wide concentration ranges from 0.1 μM to 1000 μM were found to be tolerable, however, in our study doses higher than 250 μM greatly reduced cell viability Similar effects were described in primary microglia and primary macrophage cells by using dose ranges of naltrexone from 0 to 400 μM and 1–100 μM, respectively. In addition, authors have found inhibitory effects of naltrexone on LPS-induced TNF-α in a concentration dependent manner in BV-2 cells (20 μΜ–100 μM) such as primary microglia (100 μM–400 μM) and primary macrophages (1 μM–200 μM). The authors have also shown that naltrexone administration (1 μM to 1000 μM) clearly caused inhibition of LPS-induced ROS production in a dose dependent manner, as well as inhibition of phagocytosis (10 μM–400 μM). They reported that naltrexone in doses of 200 μM inhibited LPS-induced IRF3 activation and IFN-β production in a dose dependent manner (1 μM–400 μM) [11]. The effectiveness of naltrexone was also present in a dose dependent manner in our study when observing the inhibition of mTOR signaling. In addition, our results confirm the effectiveness of (−)-naltrexone in curbing IFN-γ-induced metabolic activity, where we observed comparable effects to LPS.

Furthermore, it is important to note that the comparison between doses and efficacy in in vitro and in vivo conditions are difficult to make due to variations between the two settings. As previously said, LDN can be used to inhibit microglial activity in doses of 1–5 mg in patients [40], while significantly higher doses are used for treating alcohol-dependent patients [41]. In our setting, there was a cut-off of 250 μM, where cell viability was markedly reduced, and the same dose of 250 μM was most effective in reducing mTOR activity.

### 3.2. How Is Naltrexone Imposed into Microglia Immunometabolism—Possibly by Modulating the Phenotypic Features of Activated Microglia Related to Their Metabolic Switch?

Microglia, which are immune cells of the brain, undergo steps of differentiation and maturation triggered by environmental factors, allowing their shaping and adaptation to specific physiological function as a first line of defense [42]. Thus, microglia maintain homeostasis by assuming a diversity of phenotypes and retaining the capability to shift their functions [24].

During homeostasis, microglia are immunosuppressive and survey the environment for possible harmful stimuli. Upon stimulation, microglia can rapidly increase their phagocytic activity, switching from a branched to an ameboid-like structure. These were standardly described as M1, the classically activated pro-inflammatory phenotype, and M2, the alternatively activated anti-inflammatory phenotype [19,43,44]. The dynamic M1/M2 shift can be facilitated by endogenous as well as exogenous factors and the microglia phenotype can be reprogrammed according to the stimuli in the microenvironment. The changes in the microenvironment facilitate numerous microglial phenotypes, and it is becoming clear that the M1/M2 dichotomy does not relate to the conditions present in the complex in vivo conditions [20].

In the past few years, it has become clear that an understanding of the interaction between immune functions and metabolism can provide an insight into distinct cellular responses to challenges. Studies have observed metabolic reprogramming in macrophages in response to inflammatory stimuli, but much less is known about the similar processes in microglia [45]. Metabolic reprogramming that occurs during microglial stimulation might have an important role in the regulation of the inflammatory response and phenotype switching [24]. Further insight into the dynamics between the metabolic profile and the immune functionality of microglia could provide a better understanding of the therapeutic approach to treatment of neurological diseases [25]. Although microglia express different metabolic characteristics under various conditions, the mechanism through which the metabolic phenotype regulates microglia behavior has yet to be elucidated.

Upon activation, immune cells shift from oxidative phosphorylation to aerobic glycolysis for energy production [23,24], with the purpose of adapting to new requirements. Similar processes that might occur in the central microglia network via a link between microglial polarization and mitochondrial energy metabolism have been considered [23]. Consistent with findings in macrophages, it has been proposed that microglial metabolism also shifts with a change in microglial phenotype. It seems that anti-inflammatory M2 microglia preferentially utilize oxidative metabolism, while inflammatory changes in microglia are associated with a shift toward glycolysis and are consistent with the M1 phenotype [45]. Although it is not clear whether the metabolic switch in immune cells is necessary as a response to harmful stimuli, recent findings suggest that persistent aerobic glycolysis could negatively impact microglial function [45]. Our previous study performed on an in vitro model of CMV-infected BV-2 murine microglial cells showed extensive metabolic alteration with a shift of microglia metabolism toward glycolysis supporting the proinflammatory phenotype and activation [46]. Following the concept that the phenotypic features of microglia are associated with metabolic switching within this created M1/M2 BV-2 microglia model, we were able to test the properties of naltrexone. We used the classical markers iNOS and CD206 for the microglial phenotype to characterize the cells, with findings that naltrexone can induce increased expression of the classical M2 marker CD206 to activate ameboid microglial cells. Recent findings of CD206 expression in various diseases, namely infections and tumors, has presented a challenge to considering the anti-inflammatory microglia beneficial [47]. Although the current research landscape connects this to the capability of the tumor or the microbe to suppress the inflammatory response and “hide” from the sight of the innate immune system [48,49]. In the conditions we used, it is considered a classical anti-inflammatory marker, which we analyzed in conjunction with the metabolic analysis [50]. Our results are in line with the literature, as the CD206 positive cells had a quiescent metabolic profile, which would theoretically limit their ability to fight infections and tumors.

Furthermore, we induced a pro-inflammatory state of BV-2^10%FCS^ microglia cells by additional activation with LPS+IFN-γ inducing a highly activated glycolytic metabolic phenotype linked to high levels of ECAR and low levels of OCR (Figure 2). On the contrary, the quiescent state of microglia was characterized by high OCR and low ECAR, indicating low lactate production and high oxygen consumption in OXPHOS.

Based on this, we hypothesized that targeted drug treatment affects phenotypic switching toward oxidative metabolism underlying the protective and beneficial phenotype. Naltrexone induced an immunometabolically-suppressive phenotype of BV-2 microglia cells by converting them from a highly activated and metabolically glycolytic state toward a quiescent energetic state accompanied by oxidative metabolism due to mitochondrial adaptation (Figure 2). This metabolic shift induced by transition from glycolysis to mitochondrial oxidative metabolism is necessary for cellular adaptation and differentiation. Thus, by abolishing the highly glycolytic mode of the cells preactivated by the potent immunometabolic modulators LPS and IFN-y, naltrexone was shown to be an effective and powerful tool in metabolic reprogramming with promise in the treatment of (neuro)inflammatory conditions. Furthermore, the latest findings in the current field of viral immunology confirm naltrexone as a potential candidate for COVID-19 treatment, as it has the ability to inhibit viral infection at the level of (i) virus entry into the cell due to its molecular entities, being able to interfere with binding of the SARS-CoV-2 spike protein to ACE2, or (ii) damping the hyperinflammatory cytokine storm along with viral infectivity, by confirming its potential in this regard by suppressing LPS-induced cytokine release from pro-inflammatory macrophages [51].

### 3.3. How Is mTOR Modulated upon Metabolic State and Polarization in the Naltrexone-Induced M2 Microglia Cell Phenotype?

Cell survival in metabolically challenging environments is followed by a metabolic switch, achieved by the mTOR complex 1 (mTORC1) activation pathway wherein inputs from at least five major intracellular and extracellular cues (growth factors, stress, energy status, oxygen and amino acids) are integrated [33]. mTOR is a sensory molecule within the metabolic pathway and the function of immune cells monitoring and reprograming the features of their response [31]. Since the role of mTOR as a central integrator of cellular metabolism has been confirmed through its influence on metabolic regulation underlying phenotypic changes of immune cells [33], we attempted to gain insight into the immunometabolic profile based on mTOR-interacting proteomics by modulating mTOR activity with naltrexone.

We assessed mTOR activation indirectly through ribosomal protein S6 kinase (S6K) protein expression with the aim of estimating naltrexone-mediated regulation of mTOR in our cell model. mTORC1 activation downstream of ligand/receptor-coupled PI3K signaling leads to the phosphorylation of S6K and eukaryotic translation initiation factor 4E (eIF4E) binding proteins (4E-BPs) to stimulate translational machinery as well as energy metabolism through mitochondrial activity and biogenesis regulation [34]. A key role of the mTORC1 pathway is also the control of polarization in macrophages, which was postulated by Byles et al., and this genetic profile of macrophages associated with constitutive mTORC1 activation failed to fully upregulate the M2 anti-inflammatory program [52]. In our study, LDN administration associated with the M2 phenotype upregulated the M2 anti-inflammatory program, associated with the microglia proteomic signature related to increased expression of the total protein p70S6K and, to a lesser extent, the phosphorylated component p-p70S6K within the tested concentration range (Figure 3). Related to the proteomic feature, our data suggest that the metabolic state achieved by short-term low-dose drug application was not strictly defined as anabolic but rather balanced within the biosynthetic pathway underlying the cellular immunometabolic profile.

A possible mechanism of action of the described immunometabolic changes could be via TLR-4 receptor antagonism, which was previously described by Wang et al. [11]. There is a known connection between TLR-4 and mTOR activation in macrophages, where blocking mTOR activity was intrinsically linked to reduced TLR-4 expression [53]. Furthermore, LPS is able to induce inflammation by increasing mTOR pathway activity [54]. Taken together, our results are in line with that found by Wang et al., as blocking TLR-4 receptors could lead to a downstream reduction in mTOR activity, which appears to be necessary for pro-inflammatory metabolic microglial activation.

### 3.4. Future Prospects

According to numerous findings in this field, the dynamics of the M1/M2 shift could be of great significance for future therapeutic uses. M1 microglia act as the first line of defense against tissue injury by promoting the destruction of invading pathogens. Since M1 microglia act as pro-inflammatory agents, they are also referred to as potentially “neurotoxic microglia”. Long-lasting or unregulated, chronic inflammation and activation can lead to tissue destruction [55] and potentially have a role in promoting neuroinflammatory/neurodegenerative diseases, such as Alzheimer’s disease, Parkinson disease and multiple sclerosis [43].

Proinflammatory microglia show mitochondrial deficits and increased glycolysis, which results in reduced migration and phagocytosis and increased proinflammatory cytokine secretion [56]. It all points to immunometabolism as a possible key determinant of cell function lying at the interface between neuroinflammation and neurodegeneration [57]. Finding a medically assisted treatment that could promote the shift from constant activation of neuroinflammatory M1 to neuroprotective M2 could be of great clinical significance [19,43,58]. Moreover, repurposing naltrexone by discovery of its new properties is becoming increasingly important as a proof of concept [59] covered by the syntagm ‘an old molecule for new therapeutic approach’.

## 4. Materials and Methods

### 4.1. Cells

The BV-2 microglia cell line [60] was kindly provided by the laboratory of Professor Jasna Križ (Montreal, Canada). The cell culture protocol to maintain phenotypic and functional properties of reactive microglial cells was carried out as previously described in Blasi et al. [61]. Briefly, BV-2 cells were grown in Dulbecco’s modified Eagle’s medium (DMEM) (PAN Biotech, Aidenbach, DE) supplemented with 2 mM L-glutamine, 100 mg streptomycin and 100 U penicillin (GIBCO, Gran Island, NY, USA) (i) without fetal calf serum (FCS) or (ii) supplemented with 10% (*v*/*v*) fetal calf serum (FCS) (Thermo Fisher Scientific, Waltham, MA, USA). The cells were cultured in Petri dishes at 37 °C with 5% CO_2_ and saturated humidity. We have previously characterized the cells in other publications in relation to viral infection and antipsychotic use [46,62], and also shown that they retain expression of microglial markers (Supplementary Figure S1C,D).

### 4.2. In Vitro Cell Viability Assay

Cell viability was assessed using the Countess FL Automated Cell Counter (Thermo Fisher Scientific, Waltham, MA, USA). The semi-automated cell count was performed using the manufacturer’s instructions. The cell sample (10 μL) was mixed with 10 μL of 0.8% trypan blue stain (Sigma Aldrich, St. Louis, MO, USA). The view range and properties were optimized according to manufacturer’s instructions and the final results was confirmed with the post-analysis of the view screens. This method was validated for performing cell viability assessment [63]. The viability was assessed before each experiment as part of standard protocol and cell counting. We proceeded with the experiment if the cell viability was ≥95% in both cultivation conditions.

### 4.3. Reagents and Antibodies

Naltrexone hydrochloride (Sigma Aldrich, St. Louis, MO, USA) (−)- naltrexone stereoisomer was used for all experiments and dissolved in purified H_2_O as per the manufacturer’s instructions. Tested concentrations for naltrexone hydrochloride were 50 μM, 100 μM, 250 μM, 500 μM and 1000 μM. For immunofluorescence, we used iNOS (Thermo Fisher Scientific, Waltham, MA, USA) and CD206 (BioRad, Hercules, CA, USA) monoclonal antibodies. Secondary antibodies used were Alexa 488 goat anti-mouse IgG1, Alexa 488 and 555 goat anti-rat IgG2a and Alexa 555 goat anti-rabbit secondary reagents (all from Thermo Fisher Scientific, Waltham, MA, USA). The blue-fluorescent DNA stain DAPI was used for nuclear staining (Thermo Fisher Scientific, Waltham, MA, USA). mTOR signalling was assessed using anti-phospho-p70S6K and anti-p70S6K antibodies (Cell Signalling Technologies, MA, USA), while anti β-Actin (Sigma Aldrich, St. Louis, MO, USA) was used for protein loading control. The secondary antibody for Western blot analysis was HRP conjugated goat anti-rabbit (Cell Signalling Technologies, MA, USA). Real-time metabolism assay was performed using IFN-γ (Genentech, San Francisco, CA, USA), LPS (Sigma Aldrich, St. Louis, MO, USA) and FCCP/Oligomycin as part of the Cell Energy Phenotype Test Kit (Agilent, Santa Clara, CA, USA).

### 4.4. Immunofluorescence

Cells were grown in monolayers on glass coverslips in twenty-four well tissue-culture plates. Cells were rinsed with phosphate buffered saline (PBS) (Thermo Fisher Scientific, Waltham, MA, USA), and fixed with 4% paraformaldehyde (PFA)) (Sigma Aldrich, St. Louis, MO, USA) for 20 min. Subsequently, the cells were permeabilized with 0.1% Triton X-100 for 10 min and blocked with 2% fetal calf serum in PBS for 30 min. Labelling was done using primary and secondary antibodies for 1 h at room temperature. For analysing cell antigens, images were acquired using the fluorescent microscope Olympus BX51 (magnification of 400–1000×) (Olympus Corp, Tokyo, Japan). All immunofluorescence experiments were repeated at least three times.

### 4.5. Western Blot Analysis

Cellular extracts were prepared in RIPA lysis buffer supplemented with protease inhibitor and/or phosphatase inhibitor cocktail set (Roche, Basel, CH). Protein concentration was determined by Bradford protein assay kit (BioRad, Hercules, CA, USA), and all samples were loaded in the same concentration. The total loading dose was 20 μg per well. Proteins were separated by 12% sodium dodecyl sulphate polyacrylamide gel electrophoresis (SDS-PAGE) and blotted onto a PVDF Western blotting membrane (GE Healthcare Limited, Amersham, UK). Membranes were washed in Tris/HCl-buffered saline (TBS), blocked with 5% fat free milk for 1 h at room temperature, and incubated with primary antibodies at 4 °C overnight. After 1 h incubation at room temperature with horseradish peroxidase-coupled secondary antibodies, signals were detected with enhanced chemiluminescence Amersham ECL Prime Western Blotting Detection Reagent (GE Healthcare Limited, Amersham, UK) and scanned with an Alliance 4.0 imager (Uvitec, Cambridge, UK) according to standard methods. Western blot quantification was done in Image studio software by LI-COR Biosciences, as per the manufacturer’s instructions, considering three independent experiment samples. Statistical significance was set at *p* ≤ 0.05 values. We had issues analyzing the phopsho-p70S6K as the p70S6K substrates are rapidly phosphorylated in the samples, while p70S6K unpredictable epitopes recognized by antibodies in some cases could not be phosphorylated by p70S6K [64]. Finally, the percentage of phosphorylated (p-p) within total protein fraction (p) could be low, which led us to increase exposure to analyze the bands [65].

### 4.6. Real-Time Metabolism Assay

ECAR and OCR measurements were made using an XF-24 Extracellular Flux Analyzer (Agilent, Santa Clara, CA, USA), proven in this cell line by our group and other authors [24,62]. We used a commercial kit “Seahorse XF Cell Energy Phenotype Test Kit” to perform the cell metabolic phenotype analysis (Agilent, Santa Clara, CA, USA). The procedure simultaneously measures mitochondrial respiration and glycolysis potential by injecting pathway modulators. A utility plate containing calibrant solution (1 mL/well) together with the plates containing the injector ports and probes were placed in a CO_2_-free incubator at 37 °C overnight. The following day cells were plated at 0.2 × 10^6^ cells/well on a 24-well Seahorse plate (same-day seeding) with one well per row containing only supplemented media without cells, as a negative control. Before the assay, media was removed from cells and replaced with glucose, pyruvate and glutamine-supplemented XF assay buffer (500 mL/well) and the cell culture plate was placed in a CO_2_-free incubator for at least 1 h. Inhibitors (oligomycin and carbonyl cyanide-4-(trifluorome-thoxy) phenylhydrazone (FCCP) were added to the appropriate port of the injector plate. This plate together with the utility plate was run on the Seahorse for calibration. Once complete, the utility plate was replaced with the cell culture plate and run on the Seahorse XF-24. All cultivation conditions were done in quadruplicates to obtain the mean ECAR and OCR values provided in the results. Metabolic potential of the cells was automatically calculated using the Wave software (Agilent, Santa Clara, CA, USA), and entails the division of the stressed OCR and ECAR values with the baseline OCR and ECAR values to obtain the percentage of the cellular metabolic reserve.

### 4.7. Flow Cytometry

Cells were cultured in 12-well tissue culture plates and incubated at 37 °C and 5% CO_2_. Twenty-four hours after naltrexone administration, the cells were trypsinized and neutralized and the cellular suspension was centrifugated for 5 min at 1500 rpm. After the centrifugation, cells were resuspended and divided into flow cytometry tubes, with approximately 10^5^ cells representing one sample. Subsequently, the primary followed by corresponding secondary antibody was added to each sample. Between the incubation steps, samples were washed with FACS buffer and centrifugated for 2 min at 2000 rpm. Samples were analyzed on FACSCalibur flow cytometry (BD Biosciences, Franklin Lakes, NY, USA) using The CellQuest Pro (BDBiosciences, Franklin Lakes, NY, USA) program. The results were processed with Flowing Software (Turku, Estonia) application. We used the CD11b (Abcam, Cambridge, UK) and FITC goat α-mouse (BD Biosciences, Franklin Lakes, NY, USA) and PE rat α-mouse CD86 (BD Biosciences, Franklin Lakes, NY, USA) antibodies.

### 4.8. Statistical Analysis

Statistical analysis was performed using Statistica v13 program (TIBCO Software, Palo Alto, CA, USA). The Graph-Pad Prism program (GraphPad Software Inc., San Diego, CA, USA) and Microsoft Excel (Microsoft corp., WA, USA) were used for graphic presentation. Normal distribution was assessed using the Kolmogorov–Smirinov test, and the Shapiro–Wilk test in the case of small number of samples. Student’s t test was used to compare two independent samples. One-way ANOVA and the Tukey’s post-hoc test were used between three or more independent samples. Real-time metabolic analysis assay results are presented as mean values with standard deviations of ECAR and OCR values. Western blot quantification was done in Image studio software (LI-COR Biosciences, Lincoln, NE, USA), as per the manufacturer’s instructions, considering three independent experiment samples. Statistical significance was set at *p* ≤ 0.05 values.

## 5. Conclusions

Our innovative drug-testing model on BV-2 microglia cells has proven that naltrexone is a neuro-protective substance that induces immunometabolic alterations that might account for the M2-like anti-inflammatory phenotype based on the following:

- suppression of cell activation toward rest displaying an iNOS^low^CD206^high^ quiescence phenotype
- associated proteomic changes in mTORC1 pathway activity, analysed by p-p70S6K and p70S6K ratios
- metabolic shift from highly glycolytic to hypo-energetic cellular state, and thereby maintaining the immunometabolic balance and shape that mostly matches the protective cell microglial phenotype.

## Supplementary Materials

The following are available online at https://www.mdpi.com/article/10.3390/ijms22168429/s1.

## Abbreviations

List of abbreviations (in order of appearance).

LDN	Low-dose naltrexone
LPS	Lipopolysaccharide
IFN-γ	Interferon gamma
mTOR	Mammalian target of rapamycin
OXPHOS	oxidative phosphorylation
DMEM	Dulbecco’s modified eagle medium
FCS	Fetal calf serum
iNOS	Inducible nitric oxide synthase
CD206	Cluster of differentiation 206
OCR	Oxygen consumption rate
ECAR	Extracellular acidification rate
p70S6K	Ribosomal protein S6 kinase beta-1
p-p70S6K	Phosphorylated ribosomal protein S6 kinase beta-1
GM-CSF	granulocyte-macrophage colony-stimulating factor
TLR-4	Toll-like receptor 4
COVID-19	Coronavirus disease of 2019.
ACE2	Angiotensin-converting enzyme 2
