# Supplementary material for: Immunometabolic Modulatory Role of Naltrexone in BV-2 Microglia Cells
Source: Int J Mol Sci. 2021 Aug 5;22(16):8429. doi: 10.3390/ijms22168429 (PMC8395119; doi:10.3390/ijms22168429)
Supplement: Supplementary file 1 [file ijms-22-08429-s001.zip › ijms-1285111-supplementary.pdf]

International Journal of *Molecular Sciences*

MDPI

Supplementary

Immunometabolic Modulatory Role of Naltrexone in BV-2 Microglia Cells

Natalia Kučić [1,*], Valentino Rački [2], Roberta Šverko [3], Toni Vidović [3], Irena Grahovac [4] and Jasenka Mršić-Pelčić [5]

1 Department of Physiology and Immunology, Faculty of Medicine, University of Rijeka, Braće Branchetta 20, 51000 Rijeka, Croatia
2 Department of Neurology, Clinical Hospital Center Rijeka, University of Rijeka, Krešimirova 42, 51000 Rijeka, Croatia; valentino.racki@uniri.hr
3 Emergency Department, Clinical Hospital Center Rijeka, University of Rijeka, Krešimirova 42, 51000 Rijeka, Croatia; roberta.sverko@gmail.com (R.Š.); vidovic.toni@yahoo.com (T.V.)
4 Pharmacy Irena Grahovac, Trg I. Istarske brigade 5, 52100 Pula, Croatia; irena.grahovac@pu.t-com.hr
5 Department of Pharmacology, Faculty of Medicine, University of Rijeka, Braće Branchetta 20, 51000 Rijeka, Croatia; jasenka.mrsic.pelcic@medri.uniri.hr
* Correspondence: natalia.kucic@medri.uniri.hr; Tel.: +385-51-651-192; Fax: +385-51-675-699

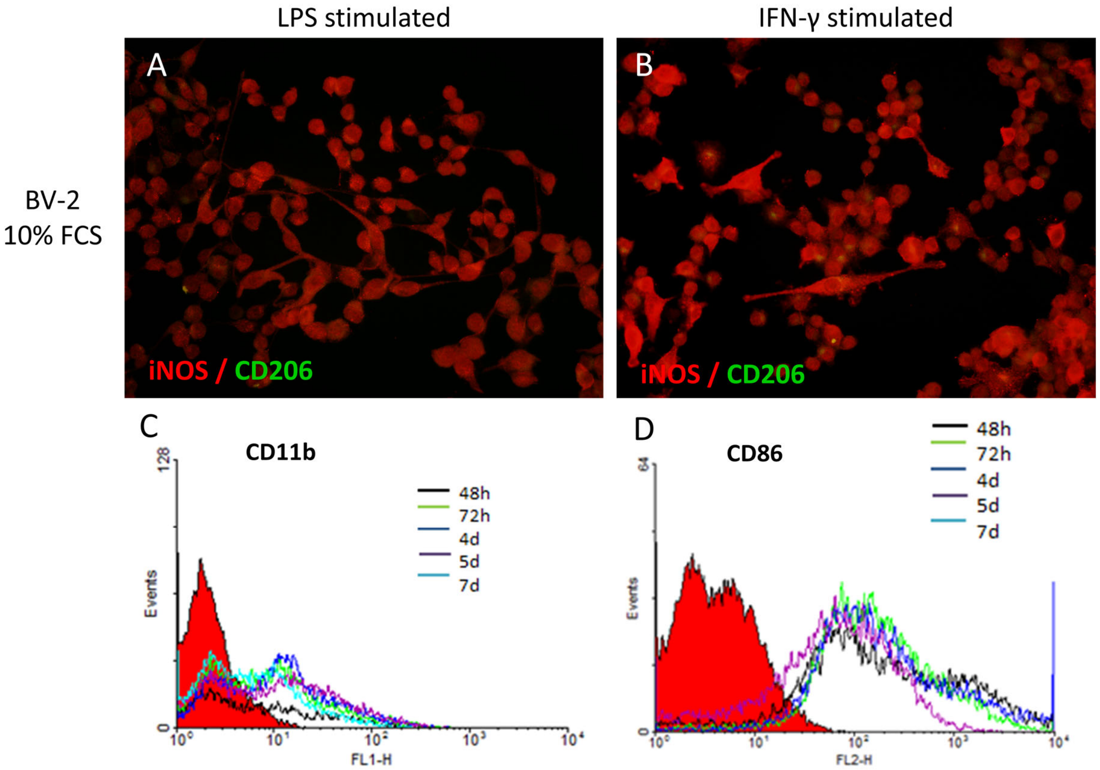

Supplementary Figure S1. Microglial phenotype upon lipopolysaccharide and intereferon gamma stimulation, and the expression of microglial phenotypic markers in cultivation conditions. Microglial cells stimulated with lipopolysaccharide (**A**) and interferon gamma (**B**) exhibit strong expressions of iNOS, while there is only minor expression of CD206 in both stimulation conditions. Cultivating BV-2 microglia cells in an extended period of time, up to 7 days, does not significantly change their expression of cell surface markers. Our cell cultures retained the expression of CD11b (**C**), confirming their lineage, while also retaining a necessary functional marker CD86 (**D**).
